# Constructing maternal morbidity – towards a standard tool to measure and monitor maternal health beyond mortality

**DOI:** 10.1186/s12884-015-0789-4

**Published:** 2016-03-02

**Authors:** Doris Chou, Özge Tunçalp, Tabassum Firoz, Maria Barreix, Veronique Filippi, Peter von Dadelszen, Nynke van den Broek, Jose Guilherme Cecatti, Lale Say, on behalf of the Maternal Morbidity Working Group

**Affiliations:** UNDP/UNFPA/UNICEF/WHO/World Bank Special Programme of Research, Development and Research Training in Human Reproduction (HRP), Department of Reproductive Health and Research, World Health Organization, Geneva, Switzerland; University of British Columbia, Vancouver, Canada; St. George’s, University of London, London, UK; London School of Hygiene & Tropical Medicine, London, UK; Centre for Maternal and Newborn Health, Liverpool School of Tropical Medicine, Liverpool, UK; UNICAMP, Faculdade de Ciências Médicas, Caixa Postal 6030, 13081-970 Campinas, SP Brazil

**Keywords:** Maternal morbidity, Definition, Measurement, Sustainable Development Goals (SDGs)

## Abstract

**Background:**

Maternal morbidity is a complex entity and its presentation and severity are on a spectrum. This paper describes the conceptualization and development of a definition for maternal morbidity, and the framework for its measurement: the maternal morbidity matrix, which is the foundation for measuring maternal morbidity, thus, the assessment tool.

**Discussion:**

We define maternal morbidity and associated disability as *“any health condition attributed to and/or complicating pregnancy and childbirth that has a negative impact on the woman’s wellbeing and/or functioning.”* A matrix of 121 conditions was generated through expert meetings, review of the International Classification of Diseases and related health problems (ICD-10), literature reviews, applying the definition of maternal morbidity and a cut-off of >0.1 % prevalence. This matrix has three dimensions: identified morbidity category, reported functioning impact and maternal history. The identification criteria for morbidity include 58 symptoms, 29 signs, 44 investigations and 35 management strategies; these criteria are aimed at recognizing the medical condition, or the functional impact/disability component that will capture the negative impact experienced by the woman.

**Summary:**

The maternal morbidity matrix is a practical framework for assessing maternal morbidity beyond near-miss. In light of the emerging attention to Universal Health Coverage (UHC) as part of the post-2015 Sustainable Development Goals (SDGs) planning, a definition and standard identification criteria are essential to measuring its extent and impact.

## Background

Improving maternal health and reducing related mortality have been key concerns of the international community as one of the eight Millennium Development Goals (MDG 5) [1]. However, maternal mortality accounts for only a small fraction of the overall burden of poor maternal health. Maternal morbidity – the health problems borne by women during pregnancy, childbirth and the postpartum period contribute to this burden. Yet, the true extent of maternal morbidity is unknown. It has been suggested that for each maternal death, 20 or 30 women suffer from morbidity; however, these calculations are not based on standard, well documented, and transparent methodologies [2, 3]. Overall, three major issues have limited valid, routine, and comparable measurements of maternal morbidity, the lack of a common definition and identification criteria, standardized assessment tools especially at primary health care level, and common indicators to measure morbidity [2]. Developing measurement criteria for the burden of pregnancy and post-partum related morbidity is crucial to the on-going elaboration of the post-2015 Sustainable Development Goals (SDGs) in light of required attention to morbidity as maternal deaths have dropped significantly over the past two decades [4].

In 2011, the World Health Organization (WHO) developed a common definition and identification criteria for very severe cases of maternal morbidity (maternal near-miss) allowing its routine measurement and monitoring, especially as a tool for assessment of the quality of care women with severe morbidity receive [5]. Such definition and criteria do not exist for less-severe cases along the continuum of maternal ill health. It is necessary to arrive at a common definition and to establish clear criteria for accurate and routine measurement of maternal morbidity in order to inform policy decisions, resource allocation and ultimately to launch an appropriate programmatic response that will also help in reducing maternal deaths, and long-term suffering and disability. This is particularly essential at the community and primary care levels, where most of the burden of maternal morbidity is believed to be reported [6, 7], yet instruments to quantify and measure it are currently lacking [2].

To fulfill the need to measure and respond to the full burden of maternal morbidity, WHO initiated a project, funded by the Bill and Melinda Gates Foundation, to improve the scientific basis for defining, measuring and monitoring maternal morbidity. This project aims to construct a definition and develop identification criteria for maternal morbidity, estimate the burden of individual causes or determining factors of maternal morbidity based on existing evidence, develop and test an assessment tool for measuring maternal morbidity in low- and middle-income countries, and develop indicators for maternal morbidity.

The project is led and carried out by a technical working group, the Maternal Morbidity Working Group (MMWG), composed of obstetricians, physicians, midwives, epidemiologists, medical anthropologists, public health professionals and patient advocates from high-, middle- and low-income countries [2]. The WHO MMWG was initially convened in April 2012. Participants were invited to join the working group based upon their known technical expertise in quantitative and qualitative maternal health research, maternal health programs, contributions to other related research initiatives or membership on WHO technical advisory groups or with potential links to this work, consumer perspective, and to ensure regional and gender balance. Where this paper reports decisions by the MMWG, these were made by consensus discussions during five WG meetings (April and August 2012, February 2013, February and October 2014) as well as interim electronic communication.

Since 2012, the MMWG has elaborated on maternal morbidity from different perspectives, and on the basis of existing evidence has agreed on a common framework for maternal morbidity. This body of work is intended to complement the maternal near-miss morbidity concept, whereby together they specify the full continuum of maternal morbidity [2]. This work will be incorporated in the 11th revision of the key standards for health conditions - the International Statistical Classification of Diseases and related health problems (ICD), further enhancing the sustainability of the outputs [8]. While doing so, publishing the development process to ensure transparency and encouraging further collaboration from researchers, clinicians, and other stakeholders have been key to the work of the MMWG.

**Fig. 1:**
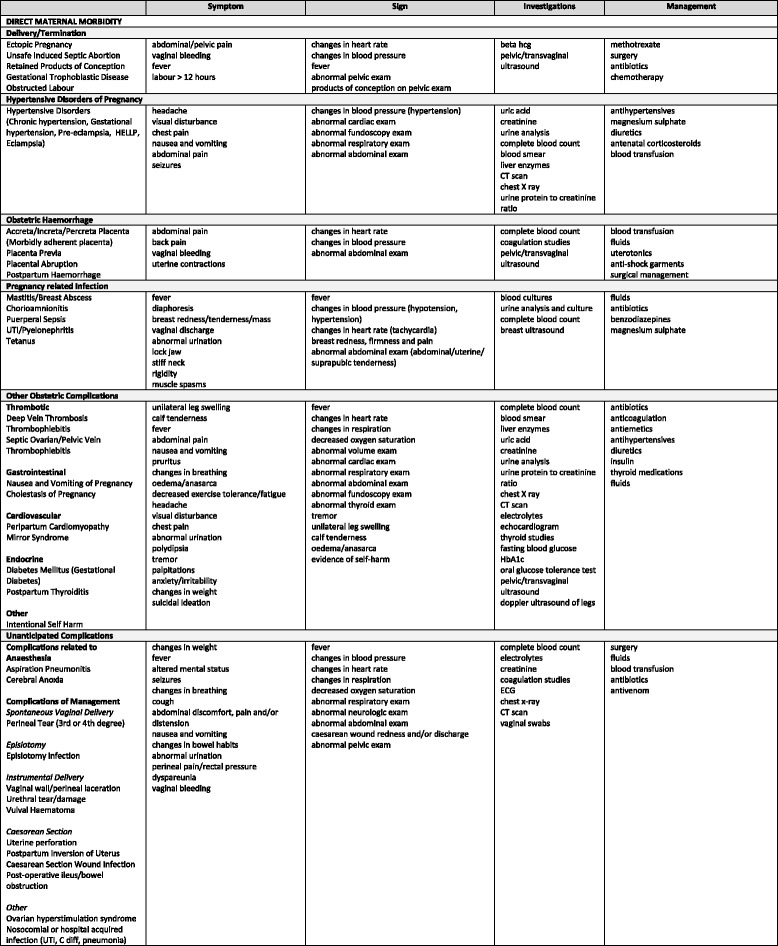
Maternal morbidity matrix, Dimension 1: SYMPTOM, SIGN, INVESTIGATIONS & MANAGEMENT (Direct Maternal Morbidity)

**Fig. 2:**
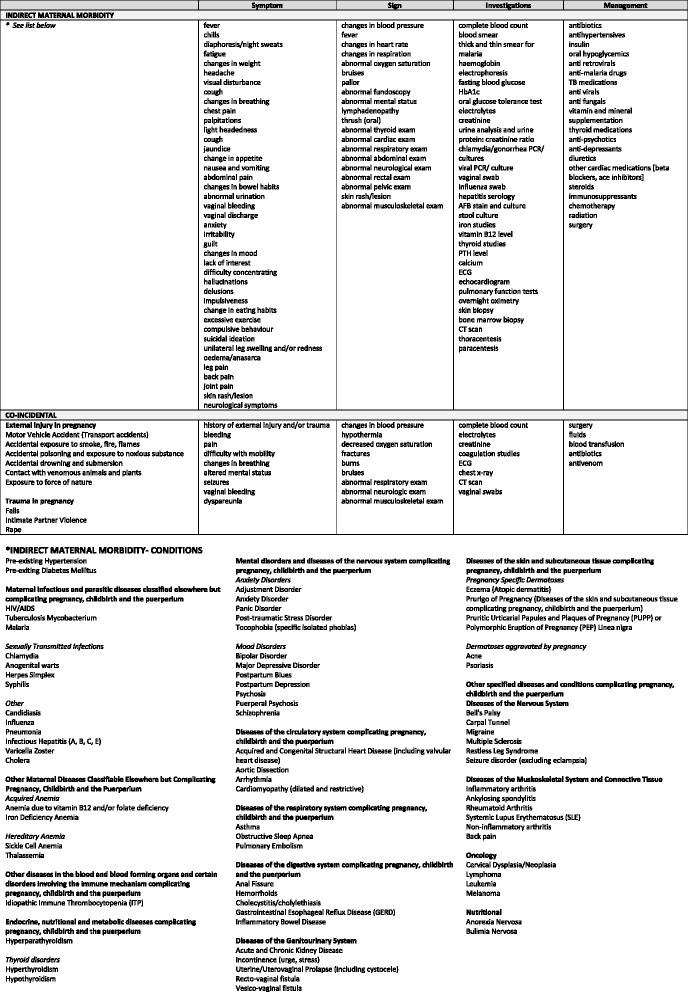
Maternal morbidity matrix, Dimension 1: SYMPTOM, SIGN, INVESTIGATIONS & MANAGEMENT (Indirect Maternal Morbidity)

**Fig. 3:**
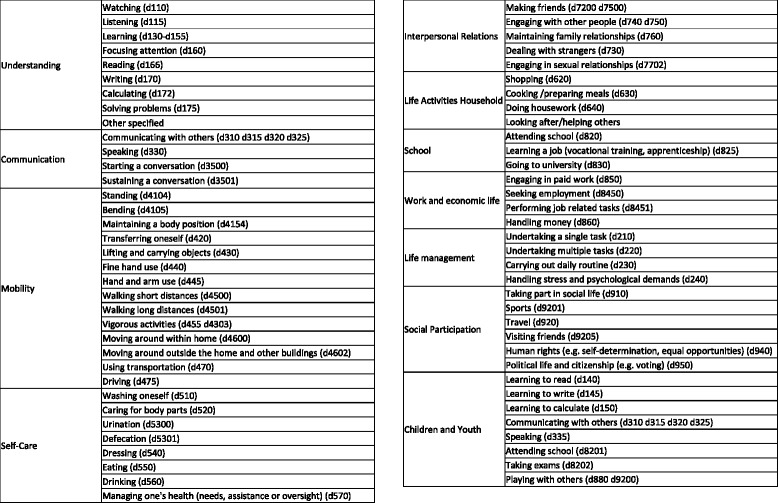
Maternal morbidity matrix, Dimension 2: Functional Impact - International Classification for Functioning and Disability (ICF) codes

**Fig. 4:**
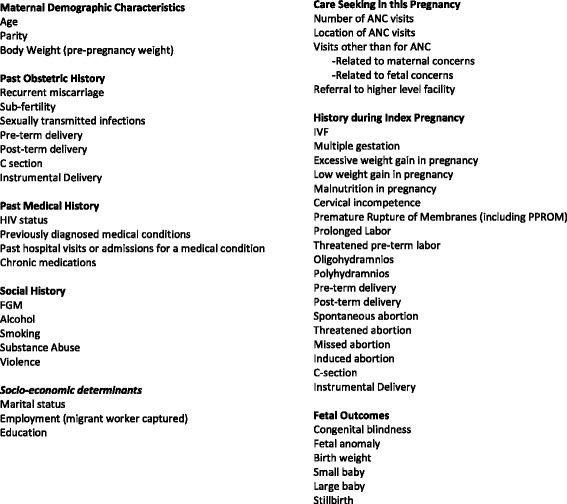
Maternal morbidity matrix, Dimension 3: Maternal History

The objective of this paper is to describe this concept, and the framework, for identifying and measuring “non-severe” maternal morbidity, and the maternal morbidity matrix (see Figs. 1,2, 3 and 4) which informed the development of a “morbidity” tool, which will be pilot tested for usability, feasibility, and fit for purpose (Please see Table 1 for an outline of the tool’s components). The "morbidity" tool is conceptualized to measure maternal morbidity in primary health care settings which have high levels of service demand [9]. Nonetheless, improved access is not enough, health services must also be of good quality [10]. Measuring morbidity can serve as an indicator of the quality of obstetric care [11, 12]. Ideally the long-term outputs of this project are to establish routine data collection on maternal morbidity to inform service provision at facility-level.

**Table 1:** Maternal morbidity draft tool components

Section 1: Personal history	Social and demographic information
	Obstetric history ( focusing on current/most recent pregnancy)
	Sexual Health
	Risk-factors/environment
Section 2: Symptoms	Disability and functioning −WHO Disability Assessment Schedule 2.0 (12-item version)
	General symptoms
	Mental health −Generalized Anxiety Disorders-7 −Personal Health Questionnaire −9
Section 3: Signs	General physical exam
	Laboratory tests and results

The MMWG deliberated the development of a community (non-health care setting) level tool to capture women who do not have regular access to medical services; however, given the time and resource constraints as well as consideration of prior research which found that women’s recall of complications has low specificity and would necessitate observation by a trained health or community worker [13, 14] the Group chose to focus its efforts on the primary facility/lowest facility level point-of-care. We discuss the development of the concept, the components of the matrix and the theoretical and methodological underpinnings for each.

## Discussion

### Concept of maternal morbidity: rationale

A standard definition for maternal morbidity does not exist nor does the literature report maternal morbidity systematically in a commonly agreed upon approach [3]. On the basis of the background scoping exercise [3], and building upon the WHO definition of health [15] and maternal mortality [16], the MMWG, by consensus, agreed on the definition for maternal morbidity and associated disability as “*any health condition attributed to and/or complicating pregnancy and childbirth that has a negative impact on the woman’s wellbeing and/or functioning.*” Heretofore, the term ‘maternal morbidity’ refers specifically to this definition of the concept. This broad definition recognizes the impact that morbidity may have on different dimensions of health, beyond physical health and seeks to encompass the totality of a woman’s sense of wellbeing. Terminology used in this manuscript can be found in Table 2 below.

**Table 2:** Definitions

Term	Definition
Maternal Death	The death of a woman while pregnant or within 42 days of termination of pregnancy, irrespective of the duration and the site of the pregnancy, from any cause related to or aggravated by the pregnancy or its management, but not from accidental or incidental causes [17]
Maternal Near-Miss	A woman who nearly died but survived a complication a complication that occurred during pregnancy, childbirth or within 42 days of termination of pregnancy [11]. Signs of organ dysfunction that follow life-threatening conditions are used to identify maternal near-misses and a set of near-miss indicators enables assessments of the quality of care provided to pregnant women [5].
Maternal morbidity and associated disability	Any health condition attributed to and/or complicating pregnancy and childbirth that has a negative impact on woman’s wellbeing and/or functioning.
Functioning	Is an umbrella term for body functions, structures, activities and participation. It denotes the negative aspects of the interaction between an individual (with a health condition) and that individual’s contextual factors (environmental and personal factors) [29].
Disability	Is an umbrella term for impairments, activity limitations and participation restrictions. It denotes the negative aspects of the interaction between an individual (with a health condition) and that individual’s contextual factors (environmental and personal factors) [29].

Based on this definition and with the goal of developing identification criteria to be embedded within a measurement tool for maternal morbidity, we initially focused on formulating and populating a matrix of conditions, not limited by the obstetrical and gynecological perspective. A number of issues were identified by the MMWG to inform the basic premises of the matrix, which members deemed necessary to include or at least consider for inclusion: 1) obstetric morbidities, 2) previous/co-existing conditions, 3) mental conditions, 4) intervention related morbidities, 5) trauma (i.e. domestic violence), and 6) cultural practices (i.e. female genital mutilation). In order to identify cases of maternal morbidity according to the agreed upon definition and to strike a balance between feasibility and utility in identification of maternal morbidity cases, we adopted a set of guiding principles to proceed with this work:

1. identification and measurement of the selected maternal conditions should be pragmatic, action oriented, evidence-based, feasible and applicable to different settings, with regional and international acceptance;
2. maternal morbidity should not be viewed as consisting only of the conditions themselves, but also their consequences; and
3. morbid conditions should be prioritized on the basis of their frequency and impact. In addition, we may focus on under- researched and neglected areas.

Balancing the tension between goals of being comprehensive and complete with usability and feasibility proved to be a challenge considering issues such as regional differences in disease incidence and prevalence, the spectrum of maternal morbidity, its occurrence, severity, duration, impact and how a morbidity affects the woman’s well-being. To focus on *“what to measure”*, we considered the role of prevalence and impact, while recognizing the need to better understand under-researched or neglected areas and the need to define what is intended by the qualifiers of “attributed to” or “complicating”. On *“how to measure”* maternal morbidity, we envisioned the development of a core module applicable to primary care settings. In either instance, the condition should be associated with a negative maternal outcome. We specified that the particular areas of interest would be the complications and/or manifestations of these conditions either during pregnancy or postpartum.

### Maternal morbidity matrix: foundations of a measurement tool

To devise identification criteria we considered categorization of different markers anatomically or by system, as was done in the development of maternal near-miss concept. However, given the particularities of less-severe pregnancy related complications, a more holistic approach was favored. Unlike maternal near-miss events, which have by definition very specific clinical, laboratory and management markers, it was understood that such markers might not be sufficient enough to identify maternal morbidity [5, 11]. As such, the maternal morbidity matrix consists of three dimensions (Figs. 1,2, 3 and 4).

Similar to the near-miss criteria, we sought to develop a set of locally relevant criteria which allow for comparisons between different settings, regions and countries. Therefore, the *first dimension* consists of the symptoms, signs, investigations and management strategies. Unlike near-miss, symptoms are included in the identification criteria of maternal morbidity, with the anticipation that they would correlate strongly with the associated disability (e.g. fatigue, shortness of breath) and thus, may be the primary reason for women to seek care. Signs are findings on physical examination and are similar to the clinical criteria of the near-miss criteria. The identification criteria also include investigations, which are broader in scope than the lab markers for the near-miss criteria, and are comprised of laboratory tests, imaging studies and diagnostic tests such as biopsies. Management strategies include treatment options like medications, surgical procedures and radiation.

Initially, the Group aimed to make the matrix as comprehensive as possible, representing both developing and developed country settings. Informed by the WHO scoping exercise on maternal morbidity [3], reviews of published literature, relevant textbooks and the WHO Application of ICD-10 to deaths during pregnancy, childbirth and the puerperium: ICD-Maternal Mortality (ICD-MM) [17], a set of conditions were selected. We considered conditions that may occur in women of reproductive age including those specific to pregnancy and postpartum. A matrix was developed, including each of these conditions and their relevant symptoms, signs, investigations and management strategies. The first version included 301 conditions, originally cross-referenced with 109 symptoms, 106 signs, 121 clinical tests and 91 management strategies [2]. At this point, the MMWG recognized the existing health care structures in low- and middle-income countries (LMICs) to balance aspirational versus pragmatic approaches. Therefore, to further consolidate the matrix, the Group developed and agreed up the following criteria:

1. conditions associated with a negative maternal outcome that are either exclusive to pregnancy, childbirth, and the postpartum state, with an estimated occurrence of >0.1 % in pregnancy; or
2. conditions that are not exclusive to pregnancy, childbirth, and postpartum but which occur more frequently during pregnancy (i.e. pregnancy is a risk factor for the disease).

The cut-off of 0.1 % for occurrence (prevalence or incidence) was deemed to be a reasonable cut-off that distinguished between very rare diseases and diseases that are more common and was informed by current estimates of disease conditions in the published literature. When evidence was unavailable, the group used a consensus mechanism based on expert opinion. Additionally, to account for regional differences in prevalence of certain conditions, the Group intends for the tool, based on the matrix, to be revised for regional implementation.

Moreover, to frame the matrix we used the precedent of ICD-MM, a special adaption of the ICD-10 intended to improve the classification of maternal mortality and morbidity [11, 17]. We grouped the domains in line with the ICD-MM, such as pregnancies with abortive outcome, obstetric hemorrhage or non-obstetric complications, with the intent of showing how data at different levels of detail may be aggregated together and to ensure continuity between the spectrum of morbidity through mortality [17]. Additionally, though it is beyond the scope of this work to revisit the definition of “direct” and “indirect” maternal mortality (and by extension, morbidity); the work of this group in reviewing the conditions aligned to each category has been informing the discussion on whether the distinction between “direct” and “indirect” remain necessary or useful. As a result of the abovementioned process, the next version of the matrix includes 121 conditions cross-referenced with all identified criteria based on the ICD-MM groupings and generated 58 symptoms, 29 signs, 44 investigations and 35 management strategies. Conditions consistent with severe maternal morbidity as manifestations of maternal near-miss were not included in this consolidated matrix as they are already identified by the maternal near-miss tool [11].

A *second dimension* is the functional impact and disability assessing the loss of physical, psychological, cognitive, social and economic functions. Key concepts related to functioning and disability as conceptualized and defined in the International Classification of Functioning, Disability and Health (ICF) are incorporated [11] thru the existing, validated tool, WHO Disability Assessment Schedule 2.0 (WHODAS 2.0) [18]. This tool covers 6 domains in line with ICF (cognition, mobility, self-care, getting along, life activities and participation) and produces standardized disability levels and profiles using a short, simple and easy to administer questionnaire [18]. In addition, preliminary findings from a systematic review on maternal morbidity and quality of life, currently in progress, will be used to refine our assessment tool to be more centered on maternal health to gauge women’s experiences.

The *third dimension* is the maternal history focusing on social and health related characteristics, which might help identify the maternal morbidity as well as influence the risk and severity of the morbidity. Some examples include socio-economic determinants, pre-existing conditions, care seeking during the pregnancy, etc. Incorporating maternal demographic characteristics, past obstetric history, history during index pregnancy and fetal outcome allows full elaboration of the “woman as a whole”. Inclusion of fetal measures in the index pregnancy appraises linkages between maternal morbidity and fetal outcomes, attesting to the irrefutable mother-baby dyad.

## Conclusions

### Time is now: implications and next steps

This body of work elaborates the first standard global definition and classification of maternal morbidity. The epidemiology of pregnancy and childbirth reflects changing demographic patterns; the latest global estimates on causes of maternal deaths demonstrate the increased role of indirect conditions in causing maternal deaths [19]. Moreover, as described in the “obstetric transition model”, with declining maternal deaths, the proportion of individuals with morbidities can only be expected to rise [20]. Beyond documenting known contributors to this changing epidemiology, e.g. the increased age at which women become pregnant [21] and co-morbidities such as obesity [22], this framework puts the focus on the women and incorporates the concepts related to disability and functioning.

Based on this concept and framework, we are in the process of developing and testing a modular set of maternal morbidity assessment tools in three country settings. This tool will have different modules depending on the time of data collection (antenatal and postnatal). The assessment tools were designed based on the matrix by thorough iterations and expert review. Whenever possible, previously validated tools and scales were employed and adapted. For example, established sexual dysfunction and mental health scales are employed as part of the 1st dimension, the WHODAS 12-item version as part of the 2nd dimension and substance abuse and intimate partner violence scales as part of the 3rd dimension [18, 23]. The results of pilot testing will direct further refinement and development of the maternal morbidity tool to ascertain the potential need for regional or country level modifications and to determine its utility in identifying morbidities and issues related to implementation of the tool. Given the paucity of data on disability from LMICs and poorer resourced areas, the interpretation of these pilots will be critical. In the future, this framework can inform a probabilistic algorithm like the Interpreting Severe Acute Maternal Morbidity (InterSAMM) to identify morbidity, which was in turn modeled after the Interpreting Verbal Autopsy - 4 (InterVA-4) to identify cause of death [24–26].

By identifying a case of maternal morbidity, either via an identified morbidity category and/or by documentation of an associated disability, we believe that the data collected by the assessment tools will have sufficient granularity and allow for disaggregation to understand what is the incidence/prevalence of a particular morbidity category, and what is the incidence/prevalence of the associated disability according to the women. Both count. In this regard, this work also coincides with the ongoing revision of the ICD. The theoretical need to “count” morbidities or clinical conditions and the women-reported outcomes inform the broader discussion of trying to understand what we measure and why.

Such discourse is particularly important as the global community debates the post-2015, Sustainable Development Goals (SDGs) agenda, with special attention to universal health coverage (UHC) as an important aspect to improve health and contribute to development of populations [27]. With regard to maternal health post-2015 goal and targets, the underlying strategies towards the Ending Preventable Maternal Mortality (EPMM) are intended to address the overall health needs of girls and women [28]. Articulated within the strategic objectives towards EPMM is the need “to address all causes of maternal mortality, reproductive and maternal morbidities, and related disabilities” and to ensure UHC for accessing care [28]. This is based upon the premise that improved information on morbidity and its lasting consequences, is also likely to expose the inter-related nature of pregnancy care to other aspects of the health sector as well as non-health sectors (e.g. environment, transportation, financing). Its most important contribution will be, however, in enhancing health system response to maternal morbidity, including strengthening evidence for the determination of packages for UHC, key means to improve access to health care and improve health of populations.

Development of a standard definition and framework for maternal morbidity is rife with challenges and the maternal morbidity matrix is a practical framework for assessing maternal morbidity beyond near-miss. In light of the emerging attention to UHC as part of the post-2015 SDGs planning, a definition and standard identification criteria are essential to measuring its extent and impact. As the international community looks at the decreases in maternal mortality, there is an urgent tandem need to define and measure maternal morbidity. Looking beyond 2015, this is an investment we cannot afford to ignore.

## Abbreviations

EPMM: Ending Preventable Maternal Mortality
ICD: International Statistical Classification of Diseases and related health problems
ICD-MM: WHO Application of ICD-10 to deaths during pregnancy, childbirth and the puerperium: ICD-Maternal Mortality
ICF: International Classification of Functioning, Disability and Health
InterSAMM: Interpreting Severe Acute Maternal Morbidity
InterVA-4: Interpreting Verbal Autopsy - 4
LMICs: Low and Middle Income Countries
MDG: Millennium Development Goal
MMWG: Maternal Morbidity Working Group
SDGs: Sustainable Development Goals
UHC: Universal Health Coverage
WHO: World Health Organization
WHODAS 2.0: WHO Disability Assessment Schedule 2.0
